# Correspondence

**Published:** 1861-02

**Authors:** A. M. Boyd

**Affiliations:** Cave Spring, Ga.


### ARTICLE III.

### Correspondence.

Mr. Editor:

Below I present you with the history of a case which I hope will interest some of the many readers of your excellent journal. Not having seen the case until after it was fully developed, and had made considerable progress, I am unable to give its early history, except as I could get it from the patient:

Mr. P., aged about fifty, of intemperate habits, had undergone much hardship, but enjoyed good health up to this time, when he supposed his liver was affected, and applied for medical aid. He was treated for dropsy. After some weeks, finding that he did not improve, he applied to a Quack, who, of course, did him no good, and on the 19th of July, 1860, I found him presenting the following symptoms: Liver much enlarged, occupying the entire hypocondriac region, and pressing upwards under the diaphragm, so as to displace the heart, and, to a considerable extent, rendering its sounds obscure. Every gland in the system, as far as it was in my power to examine them, was in a like condition. The axillary, all in the region of the neck, and the inguinal, were swollen, some of them to double the size of a man's fist, and very hard. His tongue was red and cracked; pulse rarely, if ever, below 120 per minute, quick and irregular; appetite good with impaired digestion; respiration difficult, and from 35 to 40 per minute.

I considered the case hopeless, and could promise nothing more than palliatives.

His bowels being constipated, I gave a mercurial cathartic and prescribed:

℞. Potassa Iodidii, grs. xl.
Tr. Stillingia, ℥ vii.

Dose a tablespoonful three times per day.

Potassa Bitartras, ℥ i.

Water, O. i.

Mix and take frequently through the day.

July 28th. The liver and other glands, somewhat reduced in size, and much softer; constipation relieved, having from one to three actions in twenty-four hours. These actions at first were entirely deficient in bile, but now show that the liver is acting; his pulse reduced from 120 to 90, and much more regular; tongue not so red and less cracked; says he feels considerably better than at last visit; the feet and ankles are a little swollen, and purcussion revealed the presence of some fluid in the thorax, though entirely insufficient to give cause for anxiety. Continued the same prescription and used in addition the Nitro Muriatic Acia pediluvium, and friction to all the enlarged glands and even the liver once per day.

Aug. 7th. Still improving, but very slowly; the liver and all the glands continue to decrease in size and are softer. Continue treatment, except the Bitartrate of Potassa, which is acting too freely on the kidneys.

13th. This morning I find Mr. P. complaining of acute pain in the arm, between the elbow and shoulder. On examination I find a small red spot, just above the elbow on the anterior part of the arm, not larger than a silver dollar. At first I suspected erysipelas, but the skin was not at all elevated, nor was the color bright enough. It spread rapidly towards the chest, the color undergoing no change; nor did it present any raised appearance. By the following morning it had covered the entire left side of the chest from the shoulder to the hip, and caused some tumification of the scrotum and prepuce. Neither Tr. of Iodine, Chloride of Soda, nor anything that I applied seemed to arrest or exercise any influence over its progress. The Chloride of Soda relieved the pain and itching. Finding it to be the only remedy that gave relief, I kept it constantly applied. The pulse ran up to 160 ; tongue dry and parched, with intolerable thirst ; respiration fifty to sixty and labored. At 12 o'clock I was requested to draw his urine. The scrotum and prepuce I found enormously swollen, and the whole integument of the penis black as charcoal. In attempting to introduce a catherter, the cuticle would slip off as though it had been scalded. I succeeded, however, in relieving him of his urine with considerable relief to him. In about four hours thereafter, death relieved him from his sufferings.

That the case was originally one of Hepatitis, and that this was the cause which led to the disturbance of all the other glands, I have not the shadow of a doubt ; but what was this inflammation of the skin, beginning in so small a place on the left arm, and spreading with such rapidity over one half the trunk, and that, too, in defiance of all treatment? It presented but few, if any, of the characteristics of erysipelas. In the first place, it was not red enough. The skin was not at all elevated ; the margin not well enough defined. It is my opinion—and that of an intelligent medical friend who visited the case with me—that it was erythema, but which of the almost innumerable varieties spoken of by some dermatologists, I am not able to determine. In this case I considered mercury entirely inadmissable, the patient having been severely salivated in the outset. The course under Iodine was surpassing my expectations, and I had begun to entertain some hope of recovery, or radical improvement when this eruptive complication appeared.

It is much to be regretted that the privilege of a post mortem could not be procured. It certainly would have been an interesting, if not instructive one.

A. M. BOYD.

Cave Spring, Ga., Jan. 16th, 1861.

